# Phonetic categorization in phonological lexical neighborhoods: Facilitatory and inhibitory effects

**DOI:** 10.3758/s13414-024-02931-5

**Published:** 2024-08-01

**Authors:** Yubin Zhang

**Affiliations:** Department of Linguistics, University of Southern California, Los Angeles, CA USA

**Keywords:** Phonological neighborhood, Phonetic categorization, Spoken word recognition, Lexical competition

## Abstract

Phonetic processing, whereby the bottom-up speech signal is translated into higher-level phonological representations such as phonemes, has been demonstrated to be influenced by phonological lexical neighborhoods. Previous studies show facilitatory effects of lexicality and phonological neighborhood density on phonetic categorization. However, given the evidence for lexical competition in spoken word recognition, we hypothesize that there are concurrent facilitatory and inhibitory effects of phonological lexical neighborhoods on phonetic processing. In Experiments 1 and 2, participants categorized the onset phoneme in word-nonword and nonword-word acoustic continua. The results show that the target word of the continuum exhibits facilitatory lexical influences whereas rhyme neighbors inhibit phonetic categorization. The results support the hypothesis that sublexical phonetic processing is affected by multiple facilitatory and inhibitory lexical forces in the processing stream.

## Introduction

A hallmark feature of the spoken word recognition system is that multiple phonologically similar lexical representations compete to be recognized (Luce & Pisoni, 1998; Marslen-Wilson & Welsh, 1978; McClelland & Elman, 1986; Norris, 1994; Norris & McQueen, 2008; Norris et al., 2000). Moreover, lexical information can affect phonetic processing, whereby the incoming acoustic signal is translated into higher-level phonological representations like phonemes (Fox, 1984; Ganong, 1980; Newman et al., 1997; Pitt, 1995; Rubin et al., 1976).

The current research addresses the question whether there are concurrent inhibitory and facilitatory influences of phonologically similar lexical representations on phonetic processing. In the following sections, we present a brief review of the literature on lexical competition in spoken word recognition and lexical influences on phonetic processing, followed by a summary of the possible accounts of these effects in current models of spoken word recognition and phonemic decision. Then, we turn to an introduction to the motivations and design of the current study.

### Lexical competition in spoken word recognition

Previous spoken word recognition studies on phonological neighborhood density reveal evidence for competition among phonologically similar lexical representations (Goldinger et al., 1989; Luce & Large, 2001; Luce & Pisoni, 1998; Magnuson et al., 2007; Vitevitch & Luce, 1998, 1999; Vitevitch et al., 1999).

**Fig. 1:**
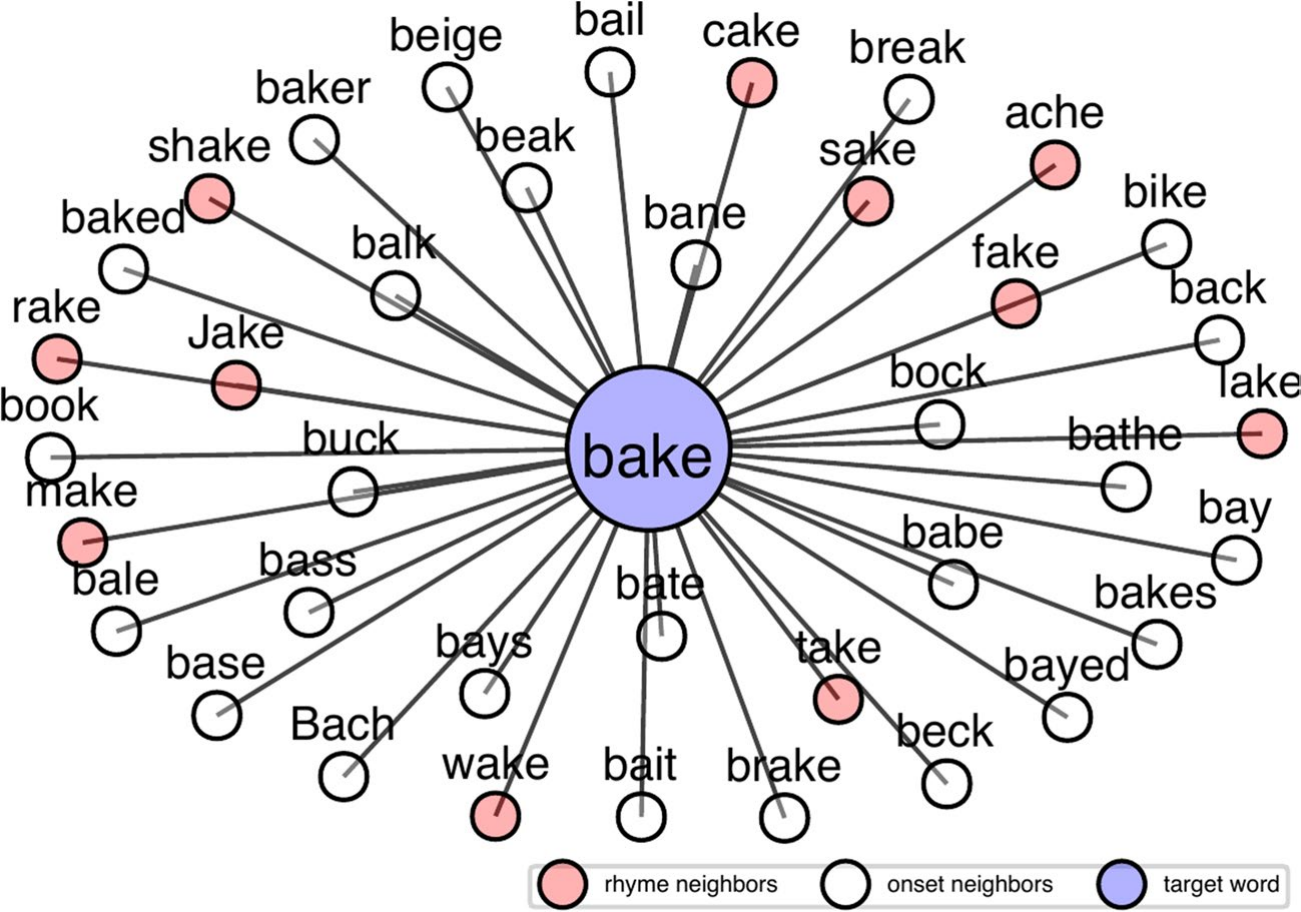
The phonological lexical neighborhood of the target word *bake* based on the “one-phoneme difference” metric. Onset neighbors are neighbors sharing the onset with the target word while rhyme neighbors are neighbors sharing the rhyme with the target word

Phonological neighborhood density is a lexical statistic to quantify phonological similarity. A frequently adopted metric for calculating phonological neighborhood density is the “one-phoneme difference” metric, i.e., words that differ from the target word by one single phoneme through substitution, addition, or deletion are the phonological neighbors of the target word. In this approach, the group of phonological neighbors constitutes a phonologically based lexical neighborhood of the target word. Phonological neighborhood density refers to the number of phonological neighbors in the neighborhood. For example, based on the *English Lexicon Project* corpus (Balota et al., 2007), the word *bake* has a relatively dense phonological neighborhood comprising 38 phonological neighbors (see Fig. 1). Among its phonological neighbors, *take*, *bike*, and *bait* can be derived by substitution of the onset /b/, the vowel /ei/, and the coda /k/ respectively. The neighbor *brake* is formed by adding a phoneme /r/ between /b/ and /ei/, while the neighbor *ache* is formed by deleting the onset phoneme /b/. The same metric can be applied to count the number of neighbors for a non-word sequence like *bab* /bæb/, which has 14 phonological neighbors. Another frequently adopted phonological neighborhood statistic is the frequency-weighted phonological neighborhood density, which is the sum of the log frequencies of all the neighbors (Newman et al., 1997; Vitevitch & Luce, 1998). Phonological neighbors can be further divided into onset and rhyme neighbors (see Fig. 1) – the neighbors sharing the same onset with a target word like *bait* are onset neighbors whereas those sharing the rhyme with a target word like *take* are rhyme neighbors (Magnuson et al., 2007; Vitevitch, 2002).

Inhibitory or competitive effects of phonological neighborhood density on spoken word recognition have been observed in a variety of tasks, such as auditory lexical decision (Luce & Pisoni, 1998; Vitevitch & Luce, 1999), auditory word naming (Luce & Pisoni, 1998; Vitevitch & Luce, 1998), same-different judgment (Luce & Large, 2001; Vitevitch & Luce, 1999), and priming (Goldinger et al., 1989; Luce et al., 2000). For example, the response latencies of lexical decision, auditory word naming, and same-different judgment have been found to be slowed down for words with more high-frequency phonological neighbors than those with fewer ones (Luce & Large, 2001; Luce & Pisoni, 1998; Vitevitch & Luce, 1998, 1999).

There is also mounting evidence that different types of phonological neighbors such as rhyme neighbors versus onset neighbors (or words sharing the first consonant–vowel (CV) sequence with the target word) exert differential influences on spoken word recognition (Allopenna et al., 1998; Magnuson et al., 2007; Vitevitch, 2002, 2007). Allopenna et al. (1998) tracked the eye movements of participants as they were instructed verbally to move one of the four objects displayed on a computer screen using a mouse (e.g., “Pick up the *beaker*; now put it below the diamond”). The three distractor objects represent three different types of competitors. The first type of competitor is a cohort competitor, which is a type of onset competitor sharing the first CV sequence with the target word (e.g., *beetle*), a rhyme competitor (e.g., *speaker*), and an unrelated competitor (e.g., *carriage*). Their results show that probability of fixations to the target and cohort competitors begins to increase and diverges from those to the rhyme and unrelated competitors early in the recognition process. As the acoustic–phonetic information of the rhyme of the target word unfolds, fixation probability to the cohort competitor decreases. At the same time, the rhyme competitor gradually receives increasing fixations, but the peak fixation probability of the rhyme competitor is still lower than that of the cohort competitor. Finally, the fixation probabilities of both cohort and rhyme competitors return to the baseline, but the fixation probability of the cohort competitor decreases more quickly and reaches the baseline earlier than the rhyme competitor.

For the influences of onset neighbor density on spoken word recognition, Vitevitch (2002) reported that words with high onset density engender longer naming and lexical decision latencies than those with low onset density. In that study, the words are matched on the total number of neighbors (phonological neighborhood density), so that higher onset density also means lower rhyme density. Thus, the high onset density/low rhyme density condition generates more inhibitory influences than the low onset density/high rhyme density condition. The result suggests that onset neighbors might play a more important role in spoken word recognition than non-onset neighbors, like rhyme neighbors.

### Lexical influences on phonetic processing

The processes at the word recognition level, like the interaction among the target word and its phonological neighbors, have been demonstrated to influence the perception or identification of sublexical phonological units (Fox, 1984; Ganong, 1980; Newman et al., 1997; Pitt, 1995; Rubin et al., 1976).

It is well established that the lexical status of speech, that is, lexicality, influences phonetic processing (Fox, 1984; Ganong, 1980; Pitt, 1995; Rubin et al., 1976). The lexicality effect of a target word has been investigated primarily in two types of phonemic decision tasks – phonetic categorization or identification and phoneme monitoring. In a phonetic categorization task, listeners make phonemic categorizations for speech stimuli in an acoustic continuum (Liberman et al., 1957). For example, to create a *voicing* continuum, like a /da-ta/ continuum, the most important acoustic parameter for this contrast – *voicing onset time* (VOT) – is manipulated to vary continuously. Participants are required to assign phonemic labels like /d/ or /t/ to stimuli in a VOT continuum.

Ganong (1980) found that listeners are more likely to categorize an ambiguous speech sound as a phoneme that makes the whole input speech sequence a real word. For example, for acoustically ambiguous tokens like those with a VOT value between the canonical /d/ and /t/, listeners are more likely to make a /d/ judgment if the rhyme is /-æʃ/ “-*ash*,” i.e., in a *dash-tash* continuum (the voiced end sequence is written on the left side of the hyphen), because *dash* is a real word but *tash* is a non-word. Similarly, if the rhyme is /-æsk/ “*-ask*,” that is, in a *dask-task* continuum, they make a /t/ response more frequently because *task* is a real word but *dask* is a non-word. A similar facilitatory lexicality effect has also been found in a phoneme monitoring task, where participants are required to detect target phonemes in the spoken materials (Cutler & Norris, 1979), for example, to detect the /d/ in *dash* as quickly as possible. Rubin et al. (1976) reported that onset phonemes in words elicit shorter response latencies than those in non-words.

In addition to the lexicality effect, there is evidence that the lexical frequency of the target word affects phonetic categorization (Connine et al., 1993). In Connine et al. (1993), one end of their word-word continua is a high-frequency word while the other end is a low-frequency word, for example, *best*-*pest* (high frequency-low frequency). They found that listeners are more likely to make a phonetic categorization response such that the whole sequence makes a high-frequency word, for example, more /b/ responses than /p/ responses in a *best*-*pest* continuum.

Another important lexical factor influencing phonetic processing is the number and frequency of phonological neighbors in the lexical neighborhoods (Boyczuk & Baum, 1999; Newman et al., 1997, 1999, 2005). Newman et al. (1997) examines the effects of phonological lexical neighborhoods on phonetic categorization. Their materials consist of VOT continua with nonwords at both the voiced and voiceless ends. For one type of the continua like *beyth-peyth*, the voiced end has higher frequency-weighted neighborhood density, but the opposite is true for the other type of the continua like *beysh-peysh* (the underlined end has higher frequency-weighted phonological density). They found that listeners’ categorization responses are shifted to the end with higher frequency-weighted phonological density, especially for relatively ambiguous stimuli in the middle of the continuum. For example, listeners make more /b/ responses for the *beyth-peyth* continuum, but more /p/ responses for the *beysh-peysh* continuum. However, these effects are not always robust and consistent. For instance, in Newman et al. (1997), the lexical neighborhood effect was found to be robust for the bilabial and velar stimuli but not for their alveolar stimuli. Nevertheless, subsequent work found similar facilitatory effects of phonological neighborhood density on phonetic categorization (Boyczuk & Baum, 1999; Newman et al., 1999, 2005).

### Modelling accounts of lexical effects

Lexical effects on target word identification and phonetic processing can be captured by spoken word recognition and phonemic decision models, like TRACE (McClelland & Elman, 1986), MERGE (Norris et al., 2000), Shortlist (Norris, 1994), and the Bayesian versions of Shortlist and MERGE – Shortlist B and Merge B (Norris & McQueen, 2008).

For lexical competition or the phonological neighborhood effect on spoken word recognition, interactive activation and competition models, like TRACE, handle it as arising from the inhibitory and excitatory interactions among processing units. The TRACE model has one input layer and three processing layers – feature, phoneme, and word layers. There are three types of connectivity implemented in the model: feedforward excitation (input- > feature, feature- > phoneme and phoneme- > word), lateral inhibition among units at each processing layer, and top-down feedback excitation (word- > phoneme and phoneme- > feature). When a spoken input is passed to the network, it leaves a “trace” at various processing levels through the excitatory and inhibitory interaction mechanisms. Words with similar phonological units are activated in parallel and inhibit each other. For example, the results of Allopenna et al. (1998) can be readily accounted for by lexical inhibition mechanisms. In that study, both onset/rhyme neighbors or competitors share phonemic units with the target word and receive excitation from the phonemic layer. However, the onset neighbors or competitors, like CV-sharing cohort competitors, exhibit phonological similarity to the target word earlier in time and thus receive earlier activation. Due to lexical competition, the early activation of the target word and onset neighbors or competitors inhibit the activation of rhyme neighbors or competitors. Consequently, onset neighbors or competitors, like CV-sharing cohort competitors, would have earlier and stronger activation than rhyme competitors. In a Bayesian framework like Shortlist B, instead of interactive activation mechanisms, lexical competition originates from path-based Bayesian evaluation (Norris & McQueen, 2008). In this model, listeners construct multiple paths of word candidate sequences given the bottom-up speech input and search for the most probable path. The final word probability, calculated based on path probabilities, can be reduced due to the presence of competitors. Simulation results show that these mechanisms successfully account for lexical competition effects in spoken word recognition (Norris & McQueen, 2008).

For the lexicality effect on phonetic processing, the TRACE model provides an account through top-down word-phoneme feedback mechanisms (McClelland & Elman, 1986). In TRACE, the perception or the identification of a phoneme depends on both bottom-up support from the input and top-down influences from the word layer. For example, when a spoken input with an ambiguous onset, like /?aʃ/ in the *dash-tash* continuum, is passed to the network, bottom-up information leads to activations of both /d/ and /t/. However, as the input signal unfolds, there is increasing evidence for the word *dash*, so that top-down feedback from this word further excites the phoneme /d/. Thus, due to the lexical feedback, the percept of the ambiguous sound is altered, and the final categorization response is also shifted. However, the MERGE model provides a different account of the lexicality effect without explicit lexical feedback mechanisms. The basic idea of MERGE is that lexical information and prelexical information are “merged” to determine a phonemic decision response. The architecture of MERGE consists of a prelexical layer like a phoneme layer, a word layer, and a phoneme decision layer. Activations at the phoneme layer excite units at the word layer, which further excite units at the phoneme decision layer. Activations at the phoneme layer can also directly spread to the phoneme decision layer. Lateral inhibition is implemented for units at the lexical and phoneme decision layers. The MERGE model assumes that participants take advantage of both prelexical and lexical information at the decision-making stage to make a phonemic response. Thus, in this model, the percept established by the bottom-up acoustic signal is not altered by lexical information. The lexicality effect occurs at a later post-perceptual phonemic decision-making stage. Alternatively, a Bayesian framework like MERGE B can account for this effect through mechanisms whereby prelexical evidence and lexical evidence are combined to update the prior probability of phonemes. Using this model, Norris and McQueen (2008) has successfully simulated a specific type of lexical effect on phonetic categorization, called the subcategorical mismatch effect.

More specifically for phonological lexical neighborhood effects on phonetic categorization, it is unclear how existing models can account for them. In models like TRACE and MERGE, these effects are also expected to arise from the parallel activation of multiple lexical representations and the interactive mechanisms among different processing units. For example, Newman et al. (1997) suggest that in an interactive activation and competition model like TRACE, the facilitatory effect of lexical neighborhoods comes from the feedback excitations from phonological neighbors at the lexical layer. In MERGE, lexical neighbor activations can merge with sublexical phonemic activations to influence the phonemic decision process. In MERGE B, lexical neighborhood effects on phonetic processing may result from the revision of phoneme prior probabilities by considering the lexical neighborhood information in the processing stream, but the exact mechanisms remain to be investigated.

### The current study

While the results of Newman et al. (1997) and subsequent work (Boyczuk & Baum, 1999; Newman et al., 1999, 2005) provide some evidence for facilitatory effects of phonological lexical neighborhoods on phonetic processing, it is not entirely clear which types of phonological neighbors cause the observed facilitatory effect. The neighborhood density calculation method employed in these studies does not distinguish different types of neighbors like neighbors with and without task-relevant onsets. For example, the neighbors of the *beyth-peyth* continuum can be further classified into three types – onset neighbors with /b/ onset like *bake*, onset neighbors with /p/ onset like *page*, and rhyme neighbors without task-relevant onsets like *faith*. The rhyme neighbors are neighbors of the non-word sequences at both the voicing and voiceless ends. As both ends of the continuum have the same number of rhyme neighbors, it is necessarily the case that the end with higher phonological neighborhood density (onset + rhyme density) would also have higher onset density. Thus, the facilitatory effect of phonological neighborhood density observed in previous studies might be largely driven by onset neighbors with task-relevant onset phonemes. As suggested by Newman et al. (1997), in interactive activation and competition models like TRACE, the excitatory lexical feedback from onset neighbors to onset phonemes might be the underlying cause.

Moreover, the possible inhibitory effects of phonological neighbors on phonetic processing remain to be established. There is a substantial amount of evidence for lexical competition (Allopenna et al., 1998; Luce & Pisoni, 1998), and nearly all spoken word recognition models incorporate some forms of lexical competition (McClelland & Elman, 1986; Norris, 1994; Norris & McQueen, 2008; Norris et al., 2000). Newman et al. (1997, 2005) suggest that the rhyme neighbors in a continuum, like *faith* for the *beyth-peyth* continuum, are neighbors of both ends of the continuum and would not contribute to the relative differences in phonological density between the two ends (or more specifically relative onset density between the two ends). However, while rhyme neighbors do not contain task-relevant onsets, they may still lead to lexical competition, hampering spoken word recognition and reducing facilitatory lexical effects. Additionally, in interactive activation and competition models, rhyme neighbors may cause the activation of task-irrelevant phonemes, interfering with the categorization of task-relevant phonemes. Thus, it is conceivable that rhyme neighbors may exhibit inhibitory lexical influences on phonetic categorization.

Building upon previous findings on lexical influences on phonetic processing (Fox, 1984; Ganong, 1980; Newman et al., 1997; Pitt, 1995) and possible predictions from current models of spoken word recognition and phonemic decision, our hypothesis is that the target word and neighbors with specific task-relevant phonemes, like the onsets in an onset-related task, may facilitate the processing of the target phoneme, while neighbors without task-relevant phonemes, like the rhyme neighbors in an onset-related task, would inhibit phonetic categorization. In the current study, we present two experiments to investigate the concurrent facilitatory and inhibitory lexical influences on phonetic processing. In Experiment 1, we investigate the influences of lexicality, lexical frequency of the target word, relative onset density between the two ends, and rhyme density on phonetic categorization using word-nonword and nonword-word acoustic continua as in Ganong (1980). In Experiment 2, we present a large-scale replication of the findings obtained in Experiment 1 using a large stimulus set.

## Experiment 1

The goals of the Experiment 1 are threefold. The primary goal is to test whether lexical influences on phonetic categorization can manifest in a single phonetic categorization task as two opposing lexical forces – onset neighbor/target word facilitation and rhyme neighbor inhibition. We predict that listeners make phonetic categorization responses such that the speech sequence makes a word (Fox, 1984; Ganong, 1980; Pitt, 1995) and the effect is larger for words with higher lexical frequency (Connine et al., 1993). Moreover, the categorization response might be biased towards the end with higher onset density (Boyczuk & Baum, 1999; Newman et al., 1997, 1999). Finally, rhyme density is expected to attenuate both the effects of lexicality and onset density on phonetic categorization, leading to the interactions between lexicality and rhyme density and between relative onset density and rhyme density.

The second goal of this experiment is to investigate the facilitatory influences of onset density when relative onset density and lexicality of the continuum are manipulated orthogonally. Previous studies cannot answer this question directly because nonword-nonword continua were used (Boyczuk & Baum, 1999; Newman et al., 1997, 1999, 2005). More specifically, we test the hypothesis that there are two additive and independent facilitatory effects of lexicality and onset density on phonetic categorization (Newman et al., 1997). If onset density and lexicality provide consistent information about the voicing of the onset phoneme, their effects should add up and greatly boost the probability of either a voicing or a voiceless response. However, if they provide inconsistent information on onset voicing, lexical neighborhood effects may offset lexicality effects. For example, for the *gift-kift* and *giss-kiss* continua, lexicality facilitation is always consistent with neighborhood density facilitation. Because the word ends have higher frequency-weighted neighborhood density, lexicality and neighborhood statistics would shift the phonetic responses toward the same voicing or voiceless end. However, for *deep-teep* and *deach-teach*, the nonword ends have higher frequency-weighted neighborhood density. The lexicality and neighborhood density effects may cancel each other out (Newman et al., 1997). Newman et al. (1997) hypothesize that the conflict between neighborhood density and lexicality effects might partially explain the absence of lexicality effect for some continua like *deep-teep* and *deach-teach*, although the lack of lexical neighborhood effects for their alveolar stimuli does not support this claim. To further test this hypothesis, we manipulate both the lexicality and relative onset density in the current experiment.

Finally, we also investigate the relative contribution of onset density and rhyme density to phonetic categorization. Based on predictions from spoken word recognition models like TRACE and previous empirical evidence from spoken word recognition experiments (Allopenna et al., 1998; Vitevitch, 2002), we predict larger effects of onset neighbors than rhyme neighbors.

### Methods

#### Materials

We constructed 20 VOT continua based on 20 monosyllabic English words with onsets /b, d, ɡ/ and /p, t, k/ (see Appendix Table 1 for the full stimulus list). The lexicality and relative frequency-weighted onset density of the continuum were manipulated orthogonally. For the ten word-nonword continua, the voiced end represents a real English word like *gas-kas* and *dog-tog*. For the other ten nonword-word continua, the voiceless end represents a word like *gake-cake* and *gase-case.* The ten continua in each lexicality condition also differ in relative frequency-weighted onset density. Half of them have higher frequency-weighted onset density for the voiced end (denser-sparser, e.g., *dog-tog* and *gase-case*, mean difference between voiced and voiceless ends = 11.30) and the other half of them have higher frequency-weighted onset density for the voiceless end (sparser-denser, e.g., *gas-kas* and *gake-cake*, mean difference = -13.79). The continua also vary in frequency-weighted rhyme density, which ranges from 5.1 to 31.9. The log-transformed lexical frequency of the target word, as a continuous variable, ranges from 0.14 to 2.92.

To calculate the neighborhood statistics, the phonological neighbors of the end-point words or nonwords were first derived using the “one-phoneme difference” metric based on the *English Lexicon Project* corpus (Brysbaert & New, 2009; Balota et al., 2007). The neighbors were further divided into onset neighbors with task-relevant onsets of both the voiced and voiceless ends and rhyme neighbors without task-relevant onsets. For onset density, the frequency-weighted onset density of both ends was calculated. Based on the onset density measures, the continua were categorized as denser-sparser and sparser-denser continua as described above. For each continuum, frequency-weighted rhyme density was also calculated. The frequency-weighted density was calculated according to the formula: $\text{FreqDensity}=\sum_{i=1}^{n}\text{log}(LexFre{q}_{i}*10)$, where $n$ is the total number of onset or rhyme neighbors. The lexical frequency of the neighbors and target word, i.e., SUBTLEX frequency per million words, was taken from the *English Lexicon Project* corpus.

The 20 VOT continua were resynthesized using the ﻿progressive cutback and replacement method (Winn, 2020). A male native speaker of American English recorded all the words and nonwords at the endpoints of the continua. Based on the recordings, the continua were resynthesized with five VOT steps (10, 35, 60, 85, and 110 ms). For each token with a voiced onset phoneme in a word-nonword or nonword-word pair, the algorithm marked the earliest signature of low-frequency periodicity as the onset of voicing. For each token with a voiceless onset phoneme, the onset of voicing was marked using the same criterion, and the onset of burst/aspiration was marked at the earliest signature of high-frequency energy. The algorithm extracted the acoustic vowel from the voiced-onset token of a specific continuum and then tracked the F0 at vowel onset. The extracted vowel from the voiced-onset token was progressively cut back from the beginning in the five steps specified (10, 35, 60, 85, and 110 ms). To control for onset F0, which can be a cue for the voicing contrast, the onset F0 was set to a constant value of 115 Hz and the consonant f0 perturbation range was set to 75 ms based on the natural tokens produced by the speaker. The algorithm reset the initial f0 of the voiced portion to 115 Hz and performed f0 interpolation over the perturbation range using PSOLA. Finally, the five-step aspiration portions extracted from the corresponding voiceless-onset token were blended into the onsets of the trimmed vowels.

#### Participants and procedure

A total of 96 participants were recruited online. Fifty participants were recruited from the student population at the University of Southern California, and they received course credits for their time. Forty-six participants were recruited from *Prolific* and they received monetary compensation for their participation. All the participants were monolingual native speakers of American English and reported no hearing, speech, or language impairments.

The participants carried out an online forced-choice onset phoneme categorization task. They were required to wear a headset for the experiment and needed to pass a headset screening test before the experiment. Upon hearing the speech stimulus, they made judgments on the onset phoneme by pressing the corresponding keys on the keyboard (one key for /b, d, ɡ/ and another key for /p, t, k/). A short practice session consisting of ten trials was provided to familiarize the participants with the procedure. The ten stimuli for the practice trials were created based on two additional five-step VOT continua (*dask-task* and *Bob-pob*), which were not included in the test trials. The responses of the practice trials were not recorded and analyzed. After the practice session, a total of 500 test trials (20 continua × 5 VOT steps × 5 repetitions) were randomly presented to the participants in one block. There was no time limit for a response. The next trial began automatically in 100 ms after a response was recorded. The whole experiment took about 30 min to complete.

#### Data analysis

A generalized linear mixed-effects model with the logit link function was fit to analyze the probability of a voiced-onset response (/b/, /d/ or /ɡ/). For the fixed-effects component, we included five main effects – *VOT*, *Lexicality* (continuum lexicality), *LogFreq* (target word log-transformed lexical frequency), *RelOnsetDen* (relative frequency-weighted onset density), *RhymeDen* (rhyme density of the continuum). *Lexicality* was simple-coded with *nonword-word* as the reference level (contrast matrix: [-1/2; 1/2]). *RelOnsetDen* was simple-coded with *sparser-denser* as the reference level (contrast matrix: [-1/2; 1/2]). The continuous variables *VOT*, *LogFreq*, and *RhymeDen* were z-standardized. The interactions between *Lexicality* and *RhymeDen*, and between *RelOnsetDen* and *RhymeDen* were included to test the inhibitory effects of rhyme neighbors. The interaction between *LogFreq* and *Lexicality* was also included to test the facilitatory effect of target word lexical frequency.

Since consonant places of articulation can affect the probability of a “voiced” response (Benki, 2001), the factor *ConsPoA* (consonant place of articulation) was also included in the model as a control variable. This variable was simple-coded with *bilabial* as the reference level (contrast matrix: [-1/3, -1/3; 2/3, -1/3; -1/3, 2/3]). For the random effects, we began with a model with random intercepts for *participant* and *continuum* (item) only. We then built a series of models with increasingly complex random effects. The fit of these models was compared using the likelihood-ratio test. The identifiable model with the best fit was reported in the results section. The by-participant random slopes for *VOT*, *Lexicality*, *RhymeDen*, *Lexicality*:*RhymeDen*, *LogFreq*, and *Lexicality*:*LogFreq*, and the by-continuum random slope for *VOT* were justified based on the results of likelihood-ratio tests.

### Results

**Fig. 2:**
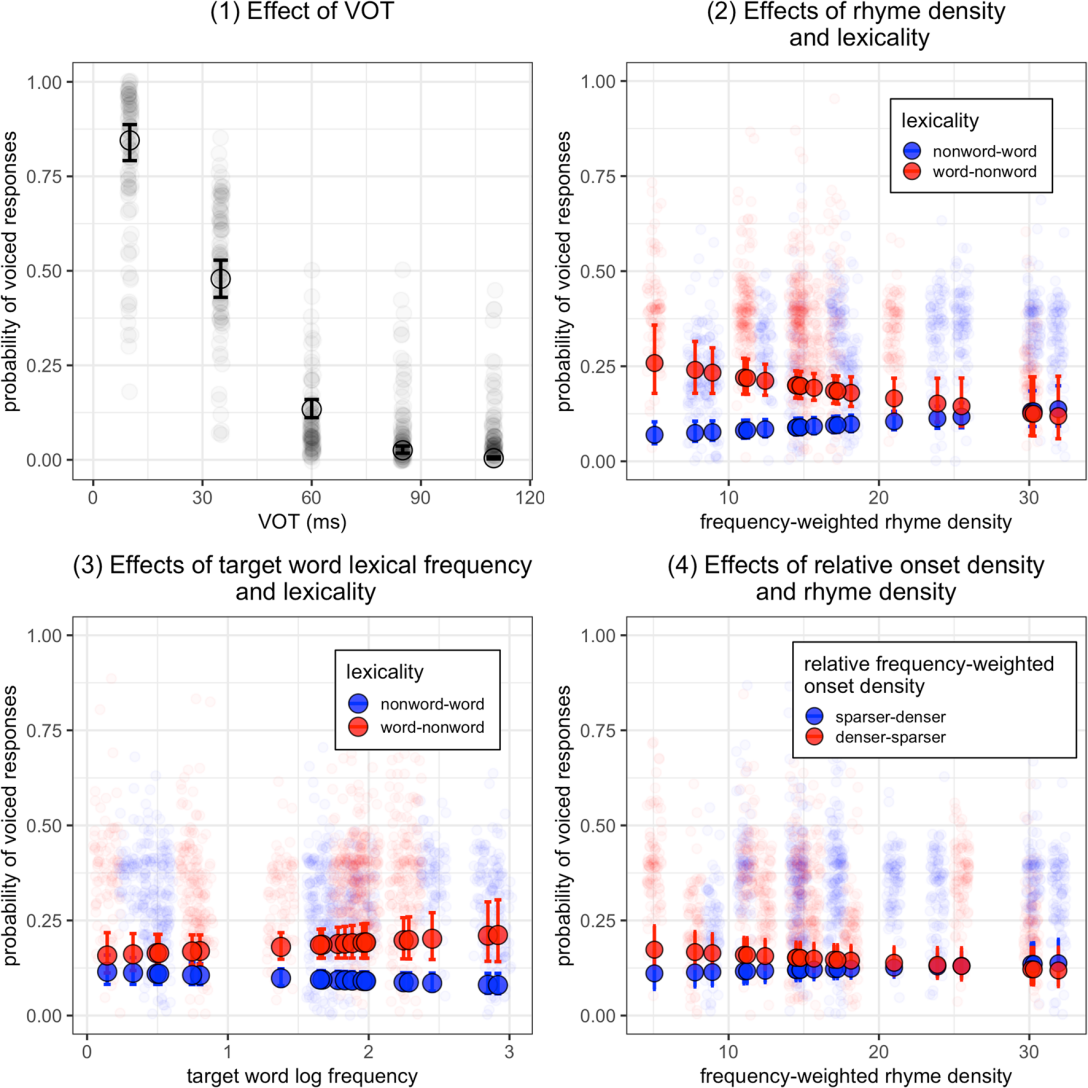
Results of Experiment 1: Probabilities of voiced-onset responses as a function of voicing onset time (VOT; panel 1), continuum lexicality (panels 2 and 3), frequency-weighted rhyme density (panels 2 and 4), target word log-transformed lexical frequency (panel 3), and relative frequency-weighted onset density (panel 4). The dots with error bars show the predicted probabilities of voiced-onset responses and standard errors based on the linear mixed-effects model. The predicted logits were transformed back to the probability space. The z-standardized continuous variables in the model were also transformed back to their original scales. The transparent dots show the proportion of voiced responses for each participant calculated based on the raw response data

The linear mixed-effects model revealed a significant main effect of *VOT* (*β* = -2.52, *p* < 0.001), indicating a lower probability of voiced-onset responses as VOT becomes larger (Fig. 2, panel 1). Moreover, a significant main effect of *Lexicality* (*β* = 0.76, *p* < 0.001) was found, suggesting a higher probability of voiced-onset responses for word-nonword continua than nonword-word continua (Fig. 2, panels 2 and 3). We also found a significant interaction between *Lexicality* and *RhymeDen* (*β* = -0.47, *p* < 0.05). The lexicality effect is smaller for continua with higher continuum rhyme density (Fig. 2, panel 2). The interaction between *Lexicality* and *LogFreq* was not significant (*β* = 0.22, *p* = 0.14), although numerically a target word with higher lexical frequency induces larger lexicality effect (Fig. 2, panel 3). We found no significant main effect of *RelOnsetDen* (*β* = 0.21, *p* = 0.26) and no significant interaction between *RelOnsetDen* and *RhymeDen* (*β* = -0.19, *p* = 0.22, Fig. 2, panel 4). There was a significant main effect of *ConsPoA* (velar versus bilabial: *β* = 0.76, *p* < 0.05). This effect suggests more voiced-onset responses for velar stops than bilabial stops.

### Discussion

In Experiment 1, consistent with previous work (Fox, 1984; Ganong, 1980; Pitt, 1995), we found a facilitatory lexicality effect of the target word of a continuum on phonetic categorization. The target word with a task-relevant phoneme facilitates the disambiguation of an unclear bottom-up acoustic signal in phonetic categorization. Furthermore, consistent with our hypothesis on rhyme neighbor inhibition, we found that frequency-weighted rhyme density inhibits the size of the lexicality effect. As the rhyme density of a continuum increases, lexical influences from the target word on phonetic categorization become smaller.

However, our results do not reveal facilitatory effects of onset neighbors with task-relevant onsets. This finding seems to contradict the results of Newman et al. (1997) and subsequent work (Boyczuk & Baum, 1999; Newman et al., 1999, 2005). The hypothesis that lexicality and onset density influences manifest as two additive and independent main effects in the behavioral output is not supported by the results. The lexical frequency effect of the target word is also not significant. Moreover, since the relative onset density effect is not significant, rhyme neighbors have been found to exhibit a larger effect on phonetic categorization than onset neighbors.

## Experiment 2

One potential limitation of Experiment 1 is the unbalanced factorial design with a limited number of specifically selected stimuli. We manipulate lexicality and relative onset density orthogonally to achieve a balanced design. However, when other confounding factors, like consonant place of articulation and vowel contexts, are considered, the design is not fully balanced. The effect size of consonant place of articulation has been reported to be relatively large in phonetic categorization (Benki, 2001). Although consonant place of articulation is included in the statistical models as a control variable, the underlying unbalanced design may still affect the model estimates and statistical inferences. Furthermore, the vowel following the consonant has been demonstrated to affect VOT perception (Rochet, 1994). Including vowels as a factor in the statistical model is potentially difficult as there are no clear criteria for systematically coding the vowel features with a small stimulus set. If we do not attempt to fully control such variables, a small stimulus set cannot ensure the randomization in the design of the experiment to address the potential confounding variables. The null effect of onset neighbors might be attributed to this potential limitation. Thus, we conduct a large-scale replication study using a large stimulus set that covers the majority of the possible monosyllabic word-nonword and nonword-word voicing pairs in the English lexicon. The goal of Experiment 2 is to test whether the findings of Experiment 1 are replicable and can generalize to a larger portion of the English vocabulary.

### Methods

#### Materials

We constructed 44 nonword-word continua and 42 word-nonword continua for Experiment 2 (see Appendix Table 2 for the full stimulus list). Like Experiment 1, the onsets of these words and nonwords have bilabial, alveolar, and velar places of articulation. For relative onset density, we first subtracted the frequency-weighted onset density of the voiceless end from that of the voiced end. Continua with positive relative onset density were coded as *denser-sparser* continua whereas those with negative relative onset density were coded as *sparser-denser* continua. Rhyme density, as a continuous variable of interest, ranges from 0 to 54.77. The neighborhood statistics and the lexical frequency of the target word were obtained in a similar way to Experiment 1.

A female native speaker of American English recorded the words and nonwords. Based on the recordings, the continua were resynthesized with five VOT steps (10, 45, 80, 115, and 150 ms). Based on the natural tokens produced by the speaker, the onset f0 was set to 260 Hz and the consonant f0 perturbation range was set to 75 ms. A similar procedure to that in Experiment 1 was used for the resynthesis.

#### Participants and procedure

A total of 91 monolingual native American English speakers without reported hearing, speech, or language impairments were recruited online. Fifty-nine of them were recruited from the student population at the University of Southern California. Course credits were granted to them as compensation for their time. Thirty-two of the participants were California residents recruited from *Prolific*. They received monetary compensation for their participation. The experimental procedure was the same as that of Experiment 1. A total of 860 test trials (20 continua × 5 VOT steps × 2 repetitions) were randomly presented to the participants in one block. The whole experiment took about 45 min to complete.

#### Data analysis

We fit a generalized linear mixed-effects model with the logit link function to analyze the probability of a voiced-onset response (/b/, /d/ or /ɡ/). The fixed-effects component included the same variables as in Experiment 1. The random-effects component included by-participant and by-continuum random intercepts. By-participant random slopes for *VOT*, *Lexicality*, *RhymeDen*, and *Lexicality*:*RhymeDen*, and the by-continuum random slope for *VOT* were also justified. The model building procedure was the same as in Experiment 1.

### Results

**Fig. 3:**
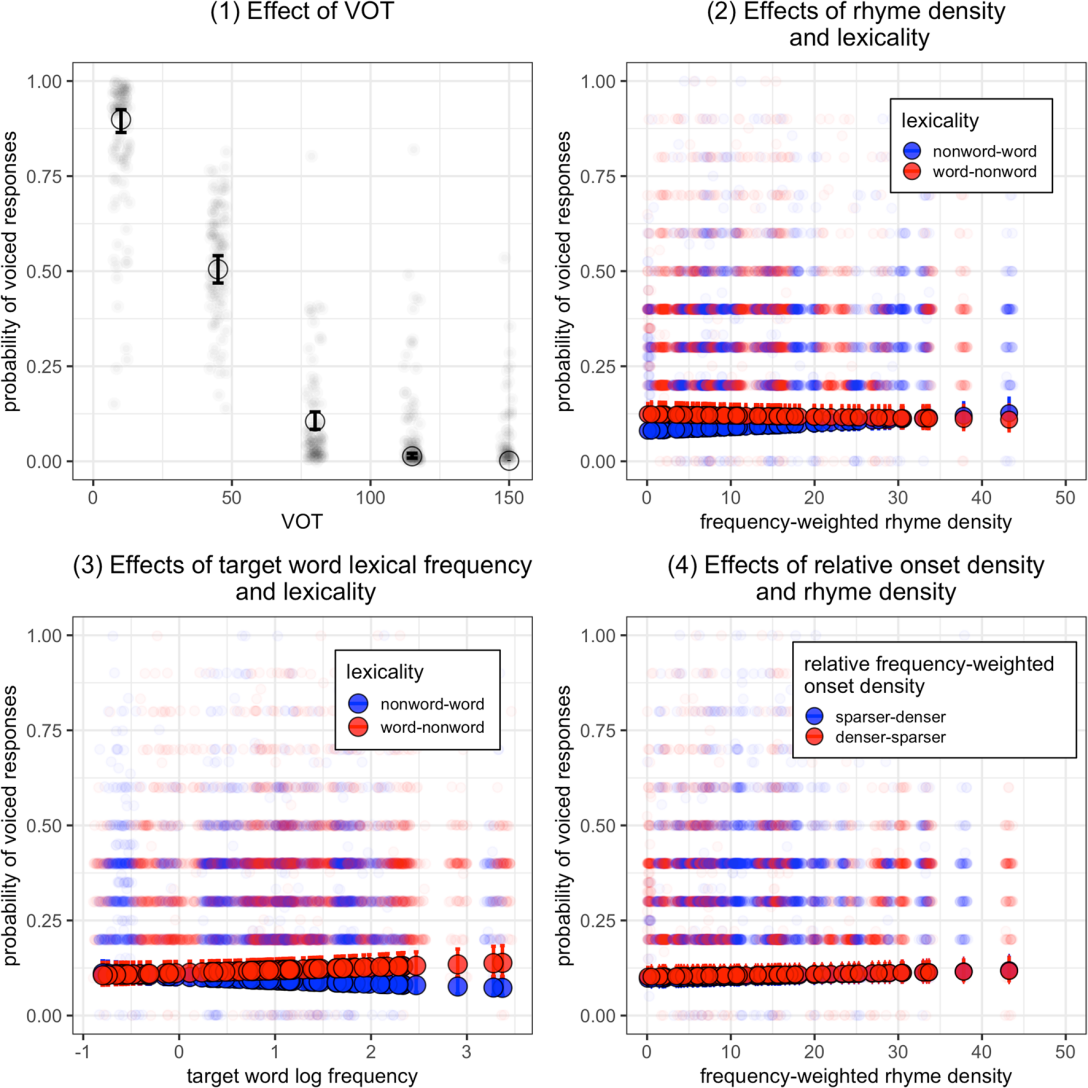
Results of Experiment 2: Probabilities of voiced-onset responses as a function of voicing onset time (VOT; panel 1), continuum lexicality (panels 2 and 3), frequency-weighted rhyme density (panels 2 and 4), target word log-transformed lexical frequency (panel 3), and relative frequency-weighted onset density (panel 4). The dots with error bars show the predicted probabilities of voiced-onset responses and standard errors based on the linear mixed-effects model. The transparent dots show the proportion of voiced responses for each participant calculated based on the raw response data

There was a significant main effect of *VOT* (*β* = -3.06, *p* < 0.001), indicating a lower probability of voiced-onset responses as VOT increases (Fig. 3, panel 1). Moreover, a significant main effect of *Lexicality* (*β* = 0.28, *p* < 0.001) was found, suggesting a higher probability of voiced-onset responses for word-nonword continua than nonword-word continua (Fig. 3, panels 2 and 3). Moreover, we found a significant interaction between *Lexicality* and *RhymeDen* (*β* = -0.16, *p* < 0.05). The negative estimate suggests an inhibitory effect of rhyme density, that is, the lexicality effect is smaller for continua with higher rhyme density (Fig. 3, panel 2). For a few continua with extremely high rhyme density, the lexicality effect seems to become negative, suggesting an opposite lexicality effect for these continua. The interaction between *Lexicality* and *LogFreq* reached significance (*β* = 0.17, *p* < 0.01). The positive estimate indicates that a target word with higher lexical frequency induces a larger lexicality effect (Fig. 3, panel 3). No significant main effect of *RelOnsetDen* (*β* = 0.04, *p* = 0.59) and no significant interaction between *RelOnsetDen* and *RhymeDen* were found (*β* = -0.02, *p* = 0.74). There was also a significant main effect of *ConsPoA* (alveolar versus bilabial: *β* = 1.15, *p* < 0.001; velar vs. bilabial: *β* = 1.37, *p* < 0.001). This effect suggests more voiced-onset responses for velar stops and alveolar stops than bilabial stops.

### Discussion

In Experiment 2, we also found that the target word with a task-relevant phoneme facilitates phonetic categorization. Additionally, the lexical frequency effect of the target word reached significance in Experiment 2. The lexical facilitation is larger when the lexical frequency of the target word becomes larger. Moreover, the rhyme neighbors of a continuum exert inhibitory influences on phonetic categorization. Higher rhyme density leads to a smaller lexicality effect of the target word. Note that it seems that the lexicality shows an opposite effect when rhyme neighbor density becomes extremely high (> 35). One possible interpretation might be that when there are many high-frequency rhyme neighbors, competition among rhyme neighbors and the target word is exceptionally strong so that participants cannot decide on a specific lexical item. Then, they might tend to make a phoneme identification response that results in a nonword, leading to an opposite lexicality effect. However, caution should be exercised when interpreting the data because there are only three continua with extremely high rhyme density (> 35). This possible cross-over pattern remains to be replicated in future investigations.

However, in both Experiment 1 and Experiment 2, we found no evidence that onset neighbors facilitate phonetic categorization. Relative onset density seems to play a minimal role in a classical phonetic categorization task using word-nonword and nonword-word continua (Fox, 1984; Ganong, 1980; Pitt, 1995). Thus, the claim that facilitatory effects of the target word and onset neighbors are independent and additive is not supported (Newman et al., 1997). The absence of a lexicality effect for some studies and some continua, for example, *deep-teep* and *deach-teach* may not be attributed to onset density. Rhyme density might be responsible for the absence of lexicality effects. For example, some of these continua like *deep-teep* have high rhyme density, which might lead to a reduced lexicality effect. Moreover, reaction time differences across studies and continua might be another contributing factor. The results of Newman et al. (1997) and Fox (1984) show some evidence that a lexicality effect occurs primarily in the intermediate and the slow reaction time partitions but not in fast responses, whereas a phonological neighborhood effect emerges primarily in the intermediate reaction time partition. In addition, other variables like biphone and triphone probability might play a role (Norris et al., 2000; Steffman & Sundara, 2024). Using a series of phonetic categorization tasks and a visual world eye-tracking task, Steffman and Sundara (2024) found independent effects of biphone probability and phonological neighborhood density on phonetic processing, but the effect of biphone probability is more robust across tasks than that of phonological neighborhood density.

Moreover, since no significant effect of onset density was found, the results do not agree with the hypothesis that onset neighbors play a more crucial role than rhyme neighbors (e.g., Allopenna et al., 1998; Vitevitch, 2002). The discrepant results might be due to the different experimental materials or task demands in different studies. For example, in Newman et al. (1997) and subsequent work (Boyczuk & Baum, 1999; Newman et al., 1999, 2005), the relative density was manipulated in nonword-nonword continua whereas we used nonword-word and word-nonword continua, which contain a dominant target word as in Ganong (1980). The facilitatory lexical effect of onset neighbors may only emerge in a phonetic categorization task using nonword-nonword continua. The theoretical implications of this point are further discussed in the next section.

## General discussion

In the current study, we conducted two experiments to investigate the facilitatory and inhibitory lexical influences on phonetic categorization. In Experiment 1 using word-nonword and nonword-word continua as in Ganong (1980), we found that the target word and rhyme neighbors exhibit facilitatory and inhibitory lexical influences, respectively. In Experiment 2 using a large stimulus set, we replicated the facilitatory and inhibitory lexical effects and found further evidence that the target word lexical frequency also modulates the facilitation. However, no hypothesized facilitatory effects of onset neighbors were found in either experiment.

The current finding is generally consistent with the larger picture of facilitatory and inhibitory effects of similar mental representations on language processing. The literature in this field has revealed various facilitatory and inhibitory neighbor effects (see Chen & Mirman, 2012, for a review). For instance, in visual word recognition, most studies reported that orthographic neighbors facilitate target word recognition (Andrews, 1997; Andrews et al., 1992). In spoken word recognition, the effects of phonological neighbors have been shown to be generally inhibitory (Luce & Pisoni, 1998; Vitevitch & Luce, 1998, 1999). Within a domain, facilitatory and inhibitory effects can occur simultaneously. For instance, in visual word recognition, while there is a growing body of evidence for facilitatory effects of orthographic neighbors, high-frequency orthographic neighbors have been shown to inhibit target word recognition (e.g., Davis et al., 2009). In semantic processing, distant semantic neighbors have been shown to exert facilitatory influences while near semantic neighbors show inhibitory influences (Mirman & Magnuson, 2008). The facilitatory and inhibitory effects of similar representations found in different domains or within the same domain have been suggested to originate from two opposing forces – the facilitation from similarity due to shared features and inhibition from competition activation (Chen & Mirman, 2012; Mirman & Magnuson, 2008). Similar representations can facilitate the processing of the target due to familiarity caused by shared forms or semantic features. Conversely, similar representations can cause competition and hamper the processing of the target. The relative weighting of these two forces has been suggested to be responsible for the various facilitatory and inhibitory neighborhood effects in different domains/tasks or in the same domain/task (Chen & Mirman, 2012).

The current study provides further evidence for concurrent facilitatory and inhibitory effects of phonological neighborhoods in a single domain and a single task. The major difference between the current study and the previous studies discussed above is that we focused on the concurrent facilitatory and inhibitory effects of neighbors at the level of sublexical phonetic processing, whereas most previous studies focused on word recognition only. We suggest that the excitatory and inhibitory principles in an interactive activation and competition model, like TRACE (McClelland & Elman, 1986) or MERGE (Norris et al., 2000) can potentially account for the facilitatory and inhibitory effects observed at the level of phonetic processing. This level is affected by both the acoustic signal and lexical information. Under this framework, the facilitatory and inhibitory phonological neighborhood effects on phonetic processing can emerge from the excitatory and inhibitory interactions within the interactive activation and competition network.

The effects of the target word on phonetic categorization should be facilitatory. The target word is the most strongly activated lexical candidate that should eventually be recognized by the spoken word recognition system. Thus, the strongest facilitatory lexical influences should come from the target word. The lexicality effect can be modelled by the top-down feedback from the word to the phoneme layer as in TRACE, or by the excitatory feedforward connection from the word layer to the phoneme decision layer as in MERGE. Moreover, the target word lexical frequency can also modulate the size of the lexical facilitation through mechanisms like changing word resting level according to lexical frequency (for other possible mechanisms, see McClelland & Elman, 1986; Strauss et al., 2007). These predictions were borne out by the current results. We found that the lexicality effect has the largest effect size compared with other phonological neighborhood effects and target word lexical frequency also modulates the size of the lexicality effect.

Phonological neighbors contain task-relevant and -irrelevant phonemes, and can exert several direct and indirect influences on phonetic processing. In an onset categorization task, neighbors that contain task-relevant phonemes are onset neighbors while neighbors that do not contain task-relevant phonemes are rhyme neighbors. For rhyme neighbors, their influences on the processing of the target onset phoneme may arise from at least two inhibitory forces. The first inhibitory force is an indirect force generated from the word level due to lexical competition. The activation of rhyme neighbors can increase overall lexical competition and hamper the target word recognition. Consequently, the target word would exert less lexical influence on phonetic processing overall. The second force may be an inhibitory force caused by the activation of task-irrelevant onsets of rhyme neighbors. In models with feedback mechanisms like TRACE, task-irrelevant onsets at the phoneme layer are activated due to lexical feedback, inhibiting the activation of the target onset. In models without feedback like MERGE, task-irrelevant onsets are activated at the phoneme decision layer and can also inhibit the target onset. Indeed, we found evidence for an inhibitory effect of rhyme neighbors in the current experiments. Newman et al. (1997) argue that rhyme neighbors do not affect their way of calculating the relative phonological neighborhood density between the two ends, so that using an overall metric of relative phonological density should be justified for their experiments. However, they neglect the possibility that rhyme neighbors shared by both ends could also affect phonetic processing. The results from the current experiments extend the previous findings (Boyczuk & Baum, 1999; Newman et al., 1997, 1999, 2005) by demonstrating that rhyme neighbors do exert influences on phonetic categorization and their effects are inhibitory in an onset identification task.

Nevertheless, models with feedback mechanisms like TRACE might still be capable of producing facilitatory effects of rhyme neighbors. This is because rhyme neighbors also send excitatory feedback to the shared rhyme unit. Increasing the number of rhyme neighbors can lead to a higher activation level of the shared rhyme unit. As a result, there might be a net excitatory effect on the target word and rhyme neighbors, and eventually an increase in the activation of the target onset phoneme. However, the current experiments reveal an inhibitory effect at the behavior level. One explanation might be that in an interactive activation and competition network, the inhibitory forces are more dominant than faciliatory forces for the current experimental settings. The simulation study by Chen and Mirman (2012) shows that the relative strengths of inhibitory and facilitatory forces in the network can vary depending on the activation levels of neighbors. A high activation level of neighbors leads to a net inhibitory effect while a low activation level of neighbors leads to a net facilitatory effect. In an onset identification task, rhyme neighbors might receive a higher level of activation than onset neighbors because the acoustic signal for the onset is predominantly unclear due to experimental manipulation. Then, the net inhibitory forces might dominate the processing stream, leading to a net inhibitory effect of rhyme neighbors at the behavioral level.

For onset neighbors, we may expect a facilitatory force influencing the processing of task-relevant onset phonemes as argued by Newman et al. (1997). The facilitatory force is caused by the parallel activation of onset neighbors, which can influence phonemic decisions via top-down word-phoneme feedback as in TRACE or direct excitatory connections from the word layer to the phoneme decision layer as in MERGE. However, in the current experiments, we did not detect effects of onset neighbors. The discrepancy between results of the current and previous experiments (Boyczuk & Baum, 1999; Newman et al., 1997, 1999, 2005) might be due to the differences in neighborhood activation dynamics caused by experimental materials or task demands. In Newman et al. (1997) and subsequent work (Boyczuk & Baum, 1999; Newman et al., 1999, 2005), the relative density was manipulated in nonword-nonword continua whereas we used nonword-word and word-nonword continua containing dominant target words as in Ganong (1980). A task using word-nonword and nonword-word continua might generally lead to stronger lexical activation than a task using nonword-nonword continua. This is because in the former case participants can actually recognize a word in some cases instead of hearing a nonword all the time. If lexical information about the target word and rhyme neighbors is heavily weighted by participants before a phonemic decision response is made, an earlier facilitatory effect of onset neighbors could possibly be diminished or even eliminated at the phonemic decision stage. It remains to be tested whether current continuous models of spoken word recognition like TRACE or MERGE can successfully account for the null result obtained in the two experiments. A model that also considers the “merge” or interaction of earlier and later lexical information in phonemic decision may better account for these findings.

The details of the neighborhood activation dynamics in different experimental settings remain to be worked out by simulation studies. Moreover, more empirical studies are needed to test the activation level hypothesis using more direct experimental manipulation. For example, phonetic processing in a rhyme (vowel) categorization task or a task that does not involve ambiguous stimuli, like phoneme monitoring, may cause different activation patterns of onset versus rhyme neighbors, possibly leading to different phonological neighborhood density effects observed. In real-world situations, different listening contexts, for example, speech recognition in normal versus adverse conditions, may also affect the patterns of phonological neighborhood density effects on phonetic processing. For example, the presence of transient environmental noise in different parts of the speech signal, like onset and rhyme, may lead to different activation levels of onset versus rhyme units. The facilitatory and inhibitory effects of different phonological neighbors in such cases may be tested by experiments that introduce noise manipulation to different parts of the acoustic signal of a word. Another promising avenue for future research is to examine the time course of facilitatory and inhibitory lexical influences using a more online task, like a visual world eye-tracking paradigm or an electroencephalography (EEG) experiment. Results from this line of research will inform current models of spoken recognition by providing further evidence on the integration or interaction of late lexical information with early one in the processing stream.

Alternatively, the lexical neighborhood effects observed in the current study may also be modelled by a Bayesian framework of phonemic decision like MERGE B (Norris & McQueen, 2008). Under this framework, lexical influences on phonetic processing can be hypothesized to come from the revision of phoneme prior probability due to the influences from the phonological lexical neighborhoods. However, it remains to be investigated whether and how such Bayesian models can account for the concurrent inhibitory and facilitatory neighborhood effects.

It should be noted that in the current study listeners generally made more voiceless responses than voiced ones. The cause of this voiceless bias is unknown. Since we still obtained a reasonably large lexicality effect of the target word as in previous studies (Fox, 1984; Ganong, 1980), especially in Experiment 1, it is less likely that the null results are due to the voiceless bias. Moreover, it is believed that lexical neighborhoods have the largest influence on phonetic categorization for ambiguous stimuli in the middle of the continuum (Newman et al., 1997). It is unclear whether the lack of stimuli eliciting 100% voiced responses at the voiced end would substantially affect the estimation of lexical effects. Thus, the effects found in the current study should still be valid. Nevertheless, future studies can replicate the findings using stimuli that elicit more balanced voiced versus voiceless responses.

## Conclusions

In the current study, we investigated the facilitatory and inhibitory influences of phonological neighborhoods on phonetic processing. Onset phoneme categorization tasks using word-nonword and nonword-word acoustic continua were administered to the participants. The results show a facilitatory lexical effect of the target word and an inhibitory lexical effect of rhyme neighbors. The results extend previous findings by showing concurrent facilitatory and inhibitory lexical effects on phonetic processing, which can be theorized as resulting from the complex interactions among processing units in an interactive activation and competition framework of spoken word recognition or phonemic decision.

## Data Availability

Available online at https://osf.io/5aqb4/

## Appendix

**Table 1:** Stimuli for experiment 1

Continuum	Log-transformed lexical frequency	Lexicality	Relative onset density	Rhyme density
gas-kas	1.83	word-nonword	sparser-denser	11.02
book-pook	2.25	word-nonword	sparser-denser	17.00
gout-kout	0.14	word-nonword	sparser-denser	14.49
dust-tust	1.38	word-nonword	sparser-denser	14.78
deep-teep	1.88	word-nonword	sparser-denser	21.01
bag-pag	1.97	word-nonword	denser-sparser	14.74
bake-pake	0.80	word-nonword	denser-sparser	30.13
dog-tog	2.29	word-nonword	denser-sparser	5.06
dove-tove	0.75	word-nonword	denser-sparser	11.23
boat-poat	1.98	word-nonword	denser-sparser	15.67
but /bʊt/-put	2.92	nonword-word	denser-sparser	7.76
duff-tough	1.96	nonword-word	denser-sparser	12.43
baw-paw	0.49	nonword-word	denser-sparser	18.14
gase-case	2.45	nonword-word	denser-sparser	25.49
bool-pool	1.67	nonword-word	denser-sparser	14.48
dack-tack	0.33	nonword-word	sparser-denser	30.29
gake-cake	1.65	nonword-word	sparser-denser	31.93
dype-type	1.78	nonword-word	sparser-denser	8.92
geep-keep	2.85	nonword-word	sparser-denser	23.89
gope-cope	0.51	nonword-word	sparser-denser	17.24

**Table 2:** Stimuli for experiment 2

Continuum	Log-transformed lexical frequency	Lexicality	Relative onset density	Rhyme density
bib-pib	-0.31	word-nonword	denser-sparser	3.73
bid-pid	1.10	word-nonword	denser-sparser	15.96
binge-pinge	-0.04	word-nonword	sparser-denser	1.45
beam-peam	0.94	word-nonword	denser-sparser	9.99
beast-peast	1.39	word-nonword	sparser-denser	10.95
beef-peef	1.29	word-nonword	denser-sparser	7.50
bean-pean	0.84	word-nonword	denser-sparser	21.95
boa-poa	0.11	word-nonword	denser-sparser	1.95
boat-poat	1.98	word-nonword	denser-sparser	15.67
bode-pode	-0.10	word-nonword	denser-sparser	24.10
bone-pone	1.42	word-nonword	denser-sparser	26.89
both-poth	2.47	word-nonword	denser-sparser	1.99
dam-tam	0.76	word-nonword	sparser-denser	23.28
dance-tance	2.17	word-nonword	denser-sparser	5.42
dash-tash	0.78	word-nonword	denser-sparser	15.34
dawn-tawn	1.41	word-nonword	sparser-denser	11.80
doff-toff	-0.80	word-nonword	sparser-denser	6.02
dog-tog	2.29	word-nonword	denser-denser	3.79
dice-tice	1.02	word-nonword	sparser-denser	14.90
diet-tiet	1.19	word-nonword	sparser-denser	1.81
dine-tine	0.63	word-nonword	denser-sparser	28.41
dive-tive	1.11	word-nonword	denser-sparser	7.95
deaf-teaf	1.16	word-nonword	denser-sparser	5.96
death-teath	2.34	word-nonword	denser-sparser	2.20
debt-tebt	1.15	word-nonword	denser-sparser	33.67
deft-teft	-0.41	word-nonword	denser-sparser	5.51
depth-tepth	0.92	word-nonword	denser-sparser	0.00
desk-tesk	1.64	word-nonword	denser-sparser	0.00
Dave-tave	1.63	word-nonword	denser-sparser	17.01
day-tay	2.90	word-nonword	sparser-denser	54.77
did-tid	3.37	word-nonword	denser-sparser	13.69
dig-tig	1.66	word-nonword	denser-sparser	14.43
disk-tisk	0.41	word-nonword	denser-sparser	4.04
dish-tish	1.06	word-nonword	denser-sparser	6.64
ditch-titch	0.90	word-nonword	sparser-denser	20.89
gag-cag	0.85	word-nonword	sparser-denser	15.87
gang-cang	1.48	word-nonword	sparser-denser	12.95
gas-cas	1.83	word-nonword	sparser-denser	11.02
gasp-casp	0.24	word-nonword	denser-sparser	0.08
gun-cun	2.33	word-nonword	sparser-denser	37.81
gush-cush	-0.15	word-nonword	sparser-denser	8.01
gust-cust	-0.33	word-nonword	sparser-denser	16.48
bick-pick	2.30	nonword-word	denser-sparser	24.74
bimp-pimp	0.94	nonword-word	sparser-denser	3.47
binch-pinch	0.79	nonword-word	denser-sparser	6.95
bink-pink	1.45	nonword-word	denser-sparser	19.93
bip-pip	0.41	nonword-word	sparser-denser	25.28
biss-piss	1.37	nonword-word	sparser-denser	14.51
beeve-peeve	-0.74	nonword-word	denser-sparser	9.15
biece-piece	2.10	nonword-word	denser-sparser	6.92
boach-poach	-0.48	nonword-word	denser-sparser	4.10
boem-poem	1.14	nonword-word	sparser-denser	0.00
boet-poet	0.96	nonword-word	denser-sparser	0.00
boke-poke	0.77	nonword-word	denser-sparser	15.75
bope-pope	1.03	nonword-word	denser-sparser	15.21
dack-tack	0.33	nonword-word	sparser-denser	28.97
dact-tact	-0.66	nonword-word	sparser-denser	18.18
dag-tag	1.14	nonword-word	denser-sparser	15.57
dask-task	1.10	nonword-word	denser-sparser	6.82
dax-tax	1.16	nonword-word	sparser-denser	20.00
dong-tong	0.36	nonword-word	sparser-denser	13.23
doss-toss	1.09	nonword-word	sparser-denser	9.20
dight-tight	1.71	nonword-word	denser-sparser	43.23
dype-type	1.78	nonword-word	sparser-denser	6.14
demp-temp	0.48	nonword-word	denser-sparser	0.52
dempt-tempt	0.40	nonword-word	sparser-denser	0.00
dend-tend	1.09	nonword-word	sparser-denser	14.49
dest-test	1.92	nonword-word	denser-sparser	30.38
dext-text	0.72	nonword-word	sparser-denser	4.41
daint-taint	-0.11	nonword-word	denser-sparser	7.44
dake-take	3.28	nonword-word	denser-sparser	27.66
dape-tape	1.84	nonword-word	denser-sparser	8.74
daste-taste	1.71	nonword-word	sparser-denser	17.66
dint-tint	-0.54	nonword-word	sparser-denser	4.82
dit-tit	0.53	nonword-word	denser-sparser	30.55
gamp-camp	1.71	nonword-word	sparser-denser	9.22
gan-can	0.59	nonword-word	sparser-denser	33.40
gast-cast	1.36	nonword-word	sparser-denser	17.62
gat-cat	1.82	nonword-word	sparser-denser	32.97
gatch-catch	2.13	nonword-word	sparser-denser	10.82
gan’t-can’t	-0.57	nonword-word	sparser-denser	7.63
gub-cub	0.32	nonword-word	sparser-denser	10.41
gud-cud	-0.60	nonword-word	sparser-denser	7.11
guff-cuff	0.76	nonword-word	sparser-denser	10.67
gup-cup	1.71	nonword-word	sparser-denser	7.04
gusp-cusp	-0.57	nonword-word	sparser-denser	0.00

*Note*: all the neighborhood density statistics are frequency-weighted

## Declarations

### Open practices statement

The data for the experiments reported here are available via the Open Science Framework at: https://osf.io/5aqb4/, and none of the experiments were preregistered.

## References

[CR1] Allopenna, P. D., Magnuson, J. S., & Tanenhaus, M. K. (1998). Tracking the time course of spoken word recognition using eye movements: Evidence for continuous mapping models. *Journal of Memory and Language,**38*(4), 419–439. 10.1006/jmla.1997.2558

[CR2] Andrews, S. (1997). The effect of orthographic similarity on lexical retrieval: Resolving neighborhood conflicts. *Psychonomic Bulletin and Review,**4*(4), 439–461. 10.3758/BF03214334

[CR3] Andrews, S., Besner, D., Chumbley, J., Grainger, J., & Masson, M. (1992). Frequency and neighborhood effects on lexical access: Lexical similarity or orthographic redundancy? *Journal of Experimental Psychology: Learning, Memory, and Cognition,**8*(2), 234–254. 10.1037/0278-7393.18.2.234

[CR4] Balota, D. A., Yap, M. J., Cortese, M. J., Hutchison, K. A., Kessler, B., Loftis, B., Neely, J. H., Nelson, D. L., Simpson, G. B., & Treiman, R. (2007). The English Lexicon Project. *Behavior Research Methods,**39*, 445–459. 17958156 10.3758/bf03193014

[CR5] Benki, J. R. (2001). Place of articulation and first formant transition pattern both affect perception of voicing in English. *Journal of Phonetics,**29*, 1–22.

[CR6] Boyczuk, J. P., & Baum, S. R. (1999). The influence of neighborhood density on phonetic categorization in aphasia. *Brain and Language,**67*(1), 46–70. 10.1006/brln.1998.2049 10191000 10.1006/brln.1998.2049

[CR7] Brysbaert, M., & New, B. (2009). Moving beyond Kučera and Francis: A critical evaluation of current word frequency norms and the introduction of a new and improved word frequency measure for American English. *Behavior Research Methods,**41*(4), 977–990. 10.3758/BRM.41.4.977 19897807 10.3758/BRM.41.4.977

[CR8] Chen, Q., & Mirman, D. (2012). Competition and cooperation among similar representations: Toward a unified account of facilitative and inhibitory effects of lexical neighbors. *Psychological Review,**119*(2), 417–430. 10.1037/a0027175.supp 22352357 10.1037/a0027175 PMC3328653

[CR9] Connine, C. M., Titone, D., & Wang, J. (1993). Auditory word recognition: Extrinsic and intrinsic effects of word frequency. *Journal of Experimental Psychology: Learning, Memory, and Cognition,**19*(1), 81–94. 10.1037/0278-7393.19.1.81 8423435 10.1037//0278-7393.19.1.81

[CR10] Cutler, A., & Norris, D. (1979). Monitoring sentence comprehension. In W. E. Cooper & E. C. T. Walker (Eds.), *Sentence processing: Psycholinguistic studies presented to Merrill Garrett* (2nd ed., pp. 113–134). Erlbaum.

[CR11] Davis, C. J., Perea, M., & Acha, J. (2009). Re(de)fining the orthographic neighborhood: The role of addition and deletion neighbors in lexical decision and reading. *Journal of Experimental Psychology: Human Perception and Performance,**35*(5), 1550-1570. 10.1037/a0014253 19803656 10.1037/a0014253

[CR12] Fox, R. A. (1984). Effect of lexical status on phonetic categorization. *Journal of Experimental Psychology: Human Perception and Performance,**10*(4), 526–540. 10.1037/0096-1523.10.4.526 6235317 10.1037//0096-1523.10.4.526

[CR13] Ganong, W. F. (1980). Phonetic categorization in auditory word perception. *Journal of Experimental Psychology: Human Perception and Performance,**6*(1), 110–125. 10.1037/0096-1523.6.1.110 6444985 10.1037//0096-1523.6.1.110

[CR14] Goldinger, S. D., Luce, P. A., & Pisoni, D. B. (1989). Priming lexical neighbors of spoken words: Effects of competition and inhibition. *Journal of Memory and Language,**28*(5), 501–518. 10.1016/0749-596X(89)90009-0 24465086 10.1016/0749-596x(89)90009-0 PMC3901307

[CR15] Liberman, A. M., Harris, K. S., Hoffman, H. S., & Griffith, B. C. (1957). The discrimination of speech sounds within and across phoneme boundaries. *Journal of Experimental Psychology,**54*(5), 358–368. 10.1037/h0044417 13481283 10.1037/h0044417

[CR16] Luce, P. A., Goldinger, S. D., Auer, E. T., & Vitevitch, M. S. (2000). Phonetic priming, neighborhood activation, and PARSYN. *Perception and Psychophysics,**62*(3), 615–625. 10.3758/BF03212113 10909252 10.3758/bf03212113

[CR17] Luce, P. A., & Large, N. R. (2001). Phonotactics, density, and entropy in spoken word recognition. *Language and Cognitive Processes,**16*(5–6), 565–581. 10.1080/01690960143000137

[CR18] Luce, P. A., & Pisoni, D. B. (1998). Recognizing spoken words: The neighborhood activation model. *Ear and Hearing,**19*(1), 1–36. 10.1097/00003446-199802000-00001 9504270 10.1097/00003446-199802000-00001 PMC3467695

[CR19] Magnuson, J. S., Dixon, J. A., Tanenhaus, M. K., & Aslin, R. N. (2007). The dynamics of lexical competition during spoken word recognition. *Cognitive Science,**31*(1), 133–156. 10.1080/03640210709336987 21635290 10.1080/03640210709336987

[CR20] Marslen-Wilson, W. D., & Welsh, A. (1978). Processing interactions and lexical access during word recognition in continuous speech. *Cognitive Psychology,**10*(1), 29–63. 10.1016/0010-0285(78)90018-X

[CR21] McClelland, J. L., & Elman, J. L. (1986). The TRACE model of speech perception. *Cognitive Psychology,**18*(1), 1–86. 10.1016/0010-0285(86)90015-0 3753912 10.1016/0010-0285(86)90015-0

[CR22] Mirman, D., & Magnuson, J. S. (2008). Attractor dynamics and semantic neighborhood density: Processing is slowed by near neighbors and speeded by distant neighbors. *Journal of Experimental Psychology: Learning Memory and Cognition,**34*(1), 65–79. 10.1037/0278-7393.34.1.65 18194055 10.1037/0278-7393.34.1.65 PMC2276160

[CR23] Newman, R. S., Sawusch, J. R., & Luce, P. A. (1997). Lexical neighborhood effects in phonetic processing. *Journal of Experimental Psychology: Human Perception and Performance,**23*(3), 873–889. 10.1037/0096-1523.23.3.873 9180048 10.1037//0096-1523.23.3.873

[CR24] Newman, R. S., Sawusch, J. R., & Luce, P. A. (1999). Underspecification and phoneme frequency in speech perception. In *Papers in Laboratory Phonology V: Acquistion and the Lexicon* (pp. 298–311).

[CR25] Newman, R. S., Sawusch, J. R., & Luce, P. A. (2005). Do postonset segments define a lexical neighborhood? *Memory and Cognition,**33*(6), 941–960. 10.3758/BF03193204 16496717 10.3758/bf03193204

[CR26] Norris, D. (1994). Shortlist: A connectionist model of continuous speech recognition. *Cognition,**52*(3), 189–234. 10.1016/0010-0277(94)90043-4

[CR27] Norris, D., & McQueen, J. M. (2008). Shortlist B: A Bayesian model of continuous speech recognition. *Psychological Review,**115*(2), 357–395. 10.1037/0033-295X.115.2.357 18426294 10.1037/0033-295X.115.2.357

[CR28] Norris, D., McQueen, J. M., & Cutler, A. (2000). Merging information in speech recognition: Feedback is never necessary. *Behavioral and Brain Sciences*. 10.1017/S0140525X00003241 11301575 10.1017/s0140525x00003241

[CR29] Pitt, M. A. (1995). The locus of the lexical shift in phoneme identification. *Journal of Experimental Psychology: Learning, Memory, and Cognition,**21*(4), 1037–1052. 10.1037/0278-7393.21.4.1037 7673866 10.1037/0278-7393.21.4.1037

[CR30] Rochet, B. L. (1994). Effects of place of articulation and vowel context on VOT production and perception for French and English stops. *Journal of the International Phonetic Association,**24*(1), 1–18. 10.1017/S0025100300004965

[CR31] Rubin, P., Turvey, M. T., & Van Gelder, P. (1976). Initial phonemes are detected faster in spoken words than in spoken nonwords. *Perception & Psychophysics,**19*(5), 394–398. 10.3758/BF03199398

[CR32] Steffman, J., & Sundara, M. (2024). Disentangling the role of biphone probability from neighborhood density in the perception of nonwords. *Language and Speech,**67*(1), 166–202. 10.1177/00238309231164982 37161351 10.1177/00238309231164982

[CR33] Strauss, T. J., Harris, H. D., & Magnuson, J. S. (2007). jTRACE: A reimplementation and extension of the TRACE model of speech perception and spoken word recognition. *Behavior Research Methods,**39*(1), 19–30. 10.3758/BF03192840 17552468 10.3758/bf03192840

[CR34] Vitevitch, M. S. (2002). Influence of onset density on spoken-word recognition. *Journal of Experimental Psychology: Human Perception and Performance,**28*(2), 270–278. 10.1037/0096-1523.28.2.270 11999854 10.1037//0096-1523.28.2.270 PMC2553695

[CR35] Vitevitch, M. S. (2007). The spread of the phonological neighborhood influences spoken word recognition. *Memory and Cognition,**35*(1), 166–175. 10.3758/BF03195952 17533890 10.3758/bf03195952 PMC2553701

[CR36] Vitevitch, M. S., & Luce, P. A. (1998). When words compete: Levels of processing in perception of spoken words. *Psychological Science,**9*(4), 325–329. 10.1111/1467-9280.00064

[CR37] Vitevitch, M. S., & Luce, P. A. (1999). Probabilistic phonotactics and neighborhood activation in spoken word recognition. *Journal of Memory and Language,**40*, 374–408. 10.1006/jmla.1998.2618

[CR38] Vitevitch, M. S., Luce, P. A., Pisoni, D. B., & Auer, E. T. (1999). Phonotactics, neighborhood activation, and lexical access for spoken words. *Brain and Language,**68*(1–2), 306–311. 10.1006/brln.1999.2116 10433774 10.1006/brln.1999.2116 PMC3466467

[CR39] Winn, M. B. (2020). Manipulation of voice onset time in speech stimuli: A tutorial and flexible Praat script. *The Journal of the Acoustical Society of America,**147*(2), 852–866. 10.1121/10.0000692 32113256 10.1121/10.0000692

